# Parents Suggest Which Indicators of Progress and Outcomes Should be Measured in Young Children with Autism Spectrum Disorder

**DOI:** 10.1007/s10803-017-3282-2

**Published:** 2017-08-31

**Authors:** Helen McConachie, Nuala Livingstone, Christopher Morris, Bryony Beresford, Ann Le Couteur, Paul Gringras, Deborah Garland, Glenys Jones, Geraldine Macdonald, Katrina Williams, Jeremy R. Parr

**Affiliations:** 1 Institute of Health and Society, Newcastle University, Sir James Spence Institute 3rd floor, Royal Victoria Infirmary, Newcastle upon Tyne, NE1 4LP UK; 2 School of Sociology, Social Policy and Social Work, Queen’s University Belfast, Belfast, Northern Ireland, UK; 3 PenCRU, Child Health Group, University of Exeter Medical School, Exeter, UK; 4 Social Policy Research Unit, University of York, York, UK; 5 Guy’s and St Thomas’ NHS Foundation Trust, London, UK; 6 National Autistic Society, North East Resource Centre, Newcastle upon Tyne, UK; 7 School of Education, University of Birmingham, Birmingham, UK; 8 Developmental Medicine, Royal Children’s Hospital, University of Melbourne and Murdoch Childrens Research Institute, Parkville, Australia; 9 Institute of Neuroscience, Newcastle University, Newcastle upon Tyne, UK; 10 Present Address: Cochrane Editorial Unit, London, UK; 11 Present Address: University of Bristol, Bristol, UK

**Keywords:** Young children, Outcomes, Measurement, Parents, Consultation

## Abstract

Evaluation of interventions for children with autism spectrum disorder (ASD) is hampered by the multitude of outcomes measured and tools used. Measurement in research with young children tends to focus on core impairments in ASD. We conducted a systematic review of qualitative studies of what matters to parents. Parent advisory groups completed structured activities to explore their perceptions of the relative importance of a wide range of outcome constructs. Their highest ranked outcomes impacted directly on everyday life and functioning (anxiety, distress, hypersensitivity, sleep problems, happiness, relationships with brothers and sisters, and parent stress). Collaboration between professionals, researchers and parents/carers is required to determine an agreed core set of outcomes to use across evaluation research.

**Electronic supplementary material:**

The online version of this article (doi:10.1007/s10803-017-3282-2) contains supplementary material, which is available to authorized users.

## Introduction

In parallel with increased early identification and diagnosis for optimal management of autism spectrum disorders (ASD), there has been an expansion of evaluations of interventions, with recent improvement in the quality of trials (Charman 2011; Oono et al. 2013). One fundamental limitation of the published literature is the lack of consensus on the most important outcomes to target and measure in evaluative research. A multitude of different tools have been inconsistently used to assess progress across a range of outcomes. These tools are rarely chosen with any theoretical justification and have variable or poor evidence of robust measurement properties (Bolte and Diehl 2013).

### What has been Measured?

Outcome measurement approaches have come from developmental theory and from clinical and disability frameworks; they have included outcomes in the broad groups of ASD symptom reduction, improvements in communication and other abilities, reduction in associated problems, gains in function, and increased participation.

To a large extent, the goals of ASD early intervention studies have been focused on improving diagnostic characteristics, such as social communication difficulties (though there has been less emphasis on interventions for restricted and repetitive behaviours and interests). Evidence is emerging that autism behavioural characteristics are underpinned by genetic, brain structure, and neuropsychological differences from typical development (Tager-Flusberg and Joseph 2003; Happé and Ronald 2008; Johnson et al. 2015; Vorstman and Ophoff 2013). Knowledge about the core impairments in ASD has been enhanced by studies of the early development of baby siblings of children with autism (so-called ‘high risk’ siblings, as they have an increased risk of developing ASD). For example, Zwaigenbaum et al. (2005, 2009) reported a range of core deficits in eye contact, visual attention, orienting to name, imitation, social interest and emotional affect, and heightened sensory-oriented behaviours. These behavioural differences have obvious consequences for the development of relationships, early language and play, which are characteristic difficulties for children with ASD. However, whether these core vulnerabilities can be changed with intervention (Dawson 2008; Green et al. 2015), whether they are useful to measure as outcomes, or whether change in them can, in turn, reduce the impact of ASD symptoms and improve activity and participation, is still under investigation.

An important advance in disability services and research is the awareness that, as well as amelioration of impairment, functioning—defined as ability to take part in daily activities—and social participation are also important outcomes as defined in the International Classification of Functioning, Disability and Health (ICF) (WHO 2007). However, the differentiation of impairment, activity and participation is not as clear in ASD as in other childhood neurodisability conditions with known neurological differences and relatively easily measured impairment (such as spasticity which limits movement in cerebral palsy). Indeed in individuals with ASD, there is evidence to suggest that the severity of autism characteristics and adaptive functioning may be unrelated (Kanne et al. 2011). The ICF conceptualisation includes an understanding that focusing an intervention on developing ‘pivotal skills’ (i.e. improving impairment) may in turn facilitate participation (Koegel et al. 2001). However, there is also evidence that activity and participation can be improved by approaches that do not directly tackle a core impairment, for example, by making environmental changes (Mesibov and Shea 2010).

One implication of both the ICF and developmental theory is that appropriate measurement of outcomes for young children with ASD should therefore include both core impairments and functional behaviours. Young children with ASD have high rates of co-occurring behaviours and problems such as sleep, faddiness about food, aggression to others, and toileting difficulties which require early intervention and advice to families on management strategies (Maskey et al. 2012; Ospina et al. 2008; Howlin et al. 2009).

As young children’s development is continuously affected by their environment, including the skills and resilience of parents and carers, it is also important to consider the impacts of interventions on the family as part of outcome measurement. Evidence is increasing that parents’ interaction style is a key mediator of child developmental outcomes in autism (e.g. Siller and Sigman 2008; Pickles et al. 2014). It is well established that parents of children with ASD are more stressed than parents of children with other disabilities (Hayes and Watson 2013) and stress can interfere with flexible parenting and interaction; yet these are the parents for whom intervention may be most helpful (e.g. Rickards et al. 2007).

### Outcomes of Importance to Parents

In recent years, there has been recognition of the crucial relevance of stakeholder engagement and participation in research to ensure that the outcomes measured are important to and appropriate for individuals affected by a condition and their carers. The field of ASD has lagged behind some other conditions in this regard (Sinha et al. 2012; Harman et al. 2015; Morris et al. 2011).

The UK Kennedy report (Kennedy 2010), ‘Getting it right for children and young people’, highlighted the need to identify a common vision between families and professionals of what services are seeking to achieve for children. Measuring outcomes that are valued by families is central to that vision. This in turn should influence what and how services are provided, and potentially which services and interventions are prioritised for research evaluation. Morris et al. (Morris et al. 2015) proposed a core suite of outcomes of care for children with neurodisability, beyond mortality and morbidity, that are valued by families and targeted by professionals. However, it is not clear whether the core outcome set would be the same when focusing only on young children with ASD.

The research described in this paper was part of a process of evidence synthesis commissioned by the UK National Institute for Health Research. The MeASURe project (Measurement in Autism Spectrum disorder Under Review) included a range of consultations alongside systematic appraisal of studies investigating the measurement properties of tools previously used in research, in order to identify (i) a potential battery of tools and outcome measures that could be recommended for use in research and clinical practice with young children with ASD (up to the age of 6 years); and (ii) research recommendations for future development of appropriate outcome measures (McConachie et al. 2015). This paper reports a scoping review of qualitative studies and consultations with parent advisory groups from the MeASURe project, aiming to identify parent views about the outcomes that are important in measuring the progress of young children with ASD.

## Methods

### Scoping Review of Qualitative Literature

#### Review Question

What child outcomes are valued by parents of children with ASD?

#### Search Strategy

A systematic search was conducted using MEDLINE, CINAHL and PsycINFO (via OVID) for papers published in English to the end of 2012. Blocks of search terms were assembled for ASD [block 1] and Qualitative Study Designs [block 2] tailored to each database (Table 1).

**Table 1:** Search strategy used for PsycInfo and adapted for the other databases

ASD terms	1	exp Pervasive Developmental Disorders/	21,449
	2	exp Developmental Disabilities/	10,206
	3	autis$.ab,ti.	24,176
	4	asperg$.ab,ti.	2493
	5	pdd.ab,ti.	1192
	6	Pervasive developmental disorder$.ab,ti.	2081
	7	kanner$.ab,ti.	345
	8	1 or 2 or 3 or 4 or 5 or 6 or 7	35,627
Qualitative study design terms	9	((“semi-structured” or semistructured or unstructured or informal or “in-depth” or indepth or “face-to-face” or structured or guide) adj3 (interview* or discussion* or questionnaire*)).ab,ti.	49,983
	10	(focus group* or qualitative or ethnograph* or fieldwork or “field work” or “key informant”).ab,ti.	95,482
	11	exp Qualitative Research/	3248
	12	exp Interviews/	9745
	13	exp Group Discussion/	3127
	14	exp Narratives/	10,680
	15	(parent$ adj3 priorit$).ab,ti.	104
	16	(desired adj1 outcome$).ab,ti.	849
	17	9 or 10 or 11 or 12 or 13 or 14 or 15 or 16	151,148
	18	8 and 17	1343

Papers were selected if they identified themes which concerned parents’ hopes for their children, experience of assessment of their children, and their priorities for intervention and education of their children, thus taking a broad approach to potential identification of ‘outcomes’. Papers were excluded if: (i) ASD was not outlined in the paper as a specific focus (e.g., if “developmental disabilities” were the conditions of interest), (ii) the paper did not involve parental responses (e.g., a paper surveying parents and teachers would be included; a paper surveying just teachers was excluded), (iii) the focus was on parents’ views and future hopes for their adult children with ASD (e.g., focus must be on parents/carers of young children), (iv) the focus was on process, i.e. the challenges parents experience in accessing services for their child, (v) the paper was not in English.

#### Selection and Data Extraction

Abstracts and titles of references retrieved by the electronic searches were screened for relevance initially by one author (NL). Two authors (CM/BB) then independently screened the longlist and full text articles and agreed upon those papers that were eligible to be included. Quotes, concepts, and any themes identified, were extracted from each paper and tabulated.

### Initial Consultation with Parent Groups

#### Aim

To explore with parents what outcomes they saw as important for measuring the progress of their young child with ASD over time.

#### Method

Parent advisory groups were recruited in the North East, South East and South West of the UK. This was conceived as public involvement in research; hence no ethics approvals were required. One group was drawn from families of disabled children who volunteer as partners in research through a Family Faculty (Morris et al. 2011). Parents of children with ASD from the Family Faculty were emailed with an invitation; 12 expressed interest and 7 participated in one or more meetings. In another site, a clinical team involves families of young children with ASD in giving advice on an ad hoc basis; here, 10 parents were invited by email and 6 participated in one or more meetings. In the third site, parents of children with ASD aged 10 years or under, and who were in touch with a voluntary organization, were invited by email; four participated in one or more meetings. Thus a total of 17 parents of children with ASD (children with a range of abilities) were involved in discussion meetings. Parents were given a shopping voucher in acknowledgement of their time and expertise and to cover travel expenses.

In each site, the session was led by two facilitators (a member of the project team and a parent involvement coordinator). The discussions were summarised and organized into themes by the parent involvement coordinators.

### Consultation with Parents About Outcome Constructs

#### Aim

To explore how parents would prioritise a broad range of outcomes.

#### Method

The first stage of the MeASURe systematic review (see Introduction above) involved the identification of outcome constructs and measures used in early intervention and longitudinal studies (McConachie et al. 2015). Using this derived list, together with the themes identified in the scoping review of qualitative research and the findings from the initial consultations with parents, a set of 62 outcome constructs was drawn up by the research team. Two members of the project team, who were not specialists in autism research (NL, GM), created ‘lay wording’ versions of the constructs (see Online Appendix 1) that were then checked for fidelity of meaning by an autism content expert (HM).

In the next round of parent group meetings, an adapted Q-sort method (Watts and Stenner 2012) was used to enable parents to rate the relative importance of the 62 outcome constructs. The task was designed to capture the priority preferences of the parents, and also enabled observation of the processes and discussions that parents had while working together to agree how to prioritise the outcome constructs.

The 62 outcome constructs were presented to each of the three parent groups on typed cards, in a random order. Parent groups were asked to discuss each construct in terms of its importance. The meaning of ‘importance’ was explained as “the importance of various things which could be measured when tracking the progress of children with autism aged up to 6 years, or in measuring the outcome of a specific preschool intervention”. Parents sorted the cards onto a ‘forced choice’ grid of the same number of boxes as constructs, in a pyramid shape on a large piece of paper. Columns on the grid were labelled for levels of importance (right to left, from +5 ‘more’ to −5 ‘less’ on an 11 point scale); columns for ratings of −1, 0 and +1 had 8 boxes, reducing in height to 3 boxes for each of −5 and +5 (boxes within columns were assumed to be of equivalent importance). Thus, because of the pyramid shape, fewer constructs could be rated at the extremes of more or less important, and most classified as moderately important. This structured process enabled the group to talk through and agree by consensus the key constructs considered by the group as ‘more important’ and ‘less important’; it was stressed that no construct was considered ‘unimportant’.

The ratings by each group were averaged.

### Discussion with Parents, Professionals and Researchers

#### Aim

To explore similarities and differences in how various stakeholders considered the importance of outcomes to be measured.

#### Method

A Discussion Day was held to bring together a range of stakeholders. The participants were three parents of children with autism; one young adult on the autism spectrum who is also a social researcher; eight professionals working in health or education (speech and language therapists, occupational therapists, paediatricians, and psychologists); and four researchers working with young children with autism.

As part of the discussion day, four small groups of individuals from similar backgrounds carried out a further adapted Q sort (similar to Consultation above) to rate the importance of a smaller set of constructs, including the 10 rated as most important by the parent groups, along with the 10 most often measured by professionals as reported in a survey conducted within the main study (McConachie et al. 2015). The day concluded with a final whole group discussion comparing the similarities and differences of views.

## Results

### Review of Qualitative Literature

**Fig. 1:**
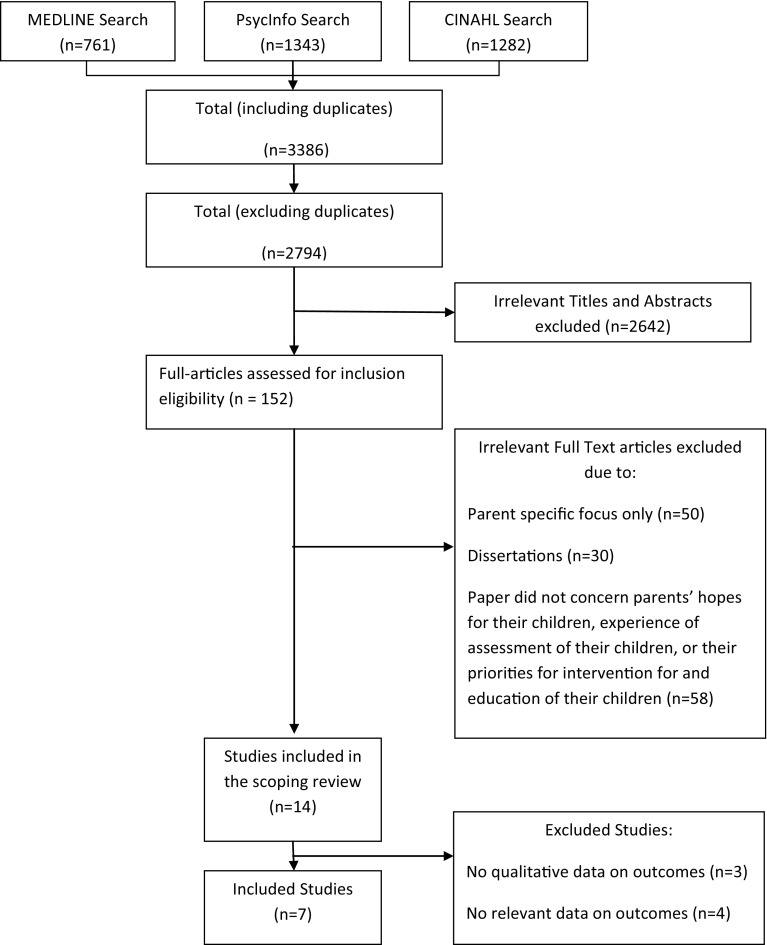
Search results of qualitative literature on outcomes valued by parents of children with ASD

Searches identified 152 papers; following title and abstract review, 14 papers were selected for retrieval of the full text. On inspection, seven papers were excluded: three did not collect qualitative data relevant to outcomes, and four contained no data on outcomes. Seven papers remained (Whitaker 2002; Beresford et al. 2006; Little and Clark 2006; Braiden et al. 2010; Serpentine et al. 2011; Auert et al. 2012; Mackintosh et al. 2012) (see Fig. 1).

Initial inspection of these seven papers showed that the quality of data was variable and ranged in focus/topic. Three papers reported data relevant to parent outcomes (Auert et al. 2012; Braiden et al. 2010; Whitaker 2002); three papers reported data relevant to child outcomes (Beresford et al. 2006; Serpentine et al. 2011; Mackintosh et al. 2012); one reported both child and parent outcomes (Little and Clark 2006). Beresford et al. also reported on data collected directly from children and young people with ASD (Beresford et al. 2006).

The age range of children and young people represented in these studies was up to 21 years. Only two studies focused specifically on younger children (Auert: 3–6 years; Whitaker: up to 5 years). Diagnoses of children and adults typically relied on parental report. Two studies focused on particular diagnostic groups (Little: Asperger syndrome; Serpentine: ‘ASD with no functional communication’); others were defined in terms of use of a particular service (Auert: speech and language therapy; Braiden: assessment and diagnosis; Whitaker: pre-school educational intervention).

Data collection methods included individual face-to-face interviews with 56 parents in total, though one study gave no sample size (Whitaker 2002), open-ended/free-text questions within postal and web-based surveys (692 parents in total) and a focus group [no sample size given (Serpentine et al. 2011)].

The quality of studies was generally poor, with low standards of reporting in relation to sampling and recruitment, data collection methods and data analysis processes. Key details in these areas were frequently missing. Given the significant limitations regarding quality and relevance, a ‘light touch’ data extraction was undertaken to identify outcomes and themes.

In terms of child outcomes, it is notable that some outcomes mentioned in the papers, and deemed ‘fundamental’ by parents, such as lack of awareness of danger—‘safety’ (Beresford et al. 2006), would not be regularly assessed and certainly not as an outcome of an early intervention trial. The parents’ and young people’s emphasis on participation outcomes (such as being “isolated from peers”, or “live a normal life”) might also not usually be measured. Constructs concerning child and parent stress, and positive mental health (Little and Clark 2006; Mackintosh et al. 2012) were identified for inclusion in the outcome constructs for further consultation.

In addition, parents often highlighted the processes of interaction with professionals, and the utility of information from assessments. Parents expected services to provide them with information and research literature; to involve them in decision-making processes; and to teach them how to deliver therapies at home. Two studies (Auert et al. 2012; Braiden et al. 2010) reported that parents “desired information relevant and applicable to their child to assist them in understanding and making sense of their own child’s presentation”. Parents also mentioned wanting to have positive times with their child: “when he is behaving well and not gearing up for a fight, he’s a very happy and pleasant child” (Little and Clark 2006). Themes from the review indicating parent priorities informed the outcome constructs included in structured discussion with subsequent parent groups (see Consultation below). For example, ‘awareness of danger’ was included as a fundamental issue affecting social inclusion.

### Initial Consultation with Parent Groups

In this initial consultation, parents discussed the outcomes that are usually assessed by professionals, and those that are not. Parents expected that professionals would focus on assessment of core features of ASD, such as social communication and social interaction impairments. However, they also suggested that the child’s skills, as well as their needs/impairments, should be acknowledged. For parents, priority areas for measurement included happiness, as well as problematic habit behaviours (such as sleep, diet and food related behaviours, sensory processing issues, toileting) and challenging behaviours and ‘meltdowns’ (such as self-harm, hitting out, anxiety, stress). Parents endorsed the importance of measuring social communication and social functioning (interacting, playing with others, playing alone, understanding and communicating) for young children. Also, for the future, parents mentioned the building blocks of learning, independence and life skills (reading and academic achievements, hobbies and sport, imagination and creativity, self-care, preparing food, getting dressed, time management, vulnerability and awareness of danger). Parents recognised that some activities/skills may not seem that important or relevant for young children, but assume significance later on in development, as their child progresses through school (e.g. making and keeping friends). Parents also mentioned difficulties they had with taking children to appointments (e.g. vaccination, dental care, buying new shoes).

In terms of the process of assessment, parents recommended the use of video recording of children’s behaviour in their various usual environments and not only in clinics. They thought this would not only improve the quality of assessments, but also allow professionals to observe changes both across settings and over time.

### Consultation with Parents About the Outcome Constructs

The full list of outcome constructs as presented to parents, with the mean rating for each across sites, is presented in Online Appendix 1. The constructs rated on average as ‘more important’ were:

Body functions/impairments: hypersensitivity, happiness, anxiety and unusual fears, distress, nonverbal ability, expressive and receptive language.

Activity level indicators: aggression, sleep problems, school readiness.

Participation: self-esteem, relationships with brothers and sisters, being bullied/rejected, no awareness of danger.

Family: parent stress.

The highest level of consistency in rating these constructs across groups was for the importance of aggression and sleep problems. Parents rated ‘happiness’ as important for all young children, but one group did not agree that this could be considered an ASD-specific measurable outcome. Children’s anxieties and distress were emphasised by parents as important outcomes to measure in that children’s emotional needs impact on the quality of life of both child and family. Parents also suggested that professionals tend to be unaware of these difficulties in the young pre-school child, i.e. before a child enters the social environment of education. In discussion, parents mentioned that they had had to learn over time about what autism is, and so had not understood at the start of assessments of their child why behaviours such as ‘joint attention skills’ were of importance, for example, to language development. The top ten average ranked constructs are presented in Table 2.

**Table 2:** Parents’ top ranked 10 constructs for measurement of progress or outcome

Parents: important areas to measure	Rank
Happiness	1
Anxiety, unusual fears	2
Discomfort with being touched, too much noise, bright lights, certain tastes, etc. (hypersensitivity)	3.5
Positive views of self (self-esteem)	3.5
Distress	5
Understanding visual information and solve problems using visual reasoning (nonverbal ability)	7
Relationships with brothers and sisters	7
Parent stress (body symptoms, poor sleep, etc)	7
Fighting, hitting others (aggression)	10
Long time to fall asleep; wake up in night	10
Experiences rejection by others; is bullied (social exclusion)	10

### Discussion with Parents, Professionals and Researchers

At the Discussion Day, four small groups were formed: one autism community group (parents and the young adult on the autism spectrum), two groups of health and education professionals, and one group of ASD researchers.

The autism community group’s ranking showed a high level of agreement with the averaged ranking of constructs done previously by the parents’ groups (Spearman rank correlation r_s_ = 0.618). Fine motor skills were rated higher in this group than by the previous parents’ groups because of the experiences of the young adult as a child. ‘Friendships’ was also rated higher, as the group reflected on the precursor skills needed by the child early on that will lead later to being able to make friendships. Aspects which affect the emotional state of the child, including sensory processing, continued to be rated highly. ‘Participates in mainstream activities’ was rated low: the autism community group thought “this means the ASD child has to adapt to the mainstream world rather than ‘mainstream’ adapting/understanding/respecting ASD needs”. They also gave a low rating to ‘not cooperating, throwing, spitting, won’t sit (maladaptive behaviour)’ since they considered it the role of adults (parents, education and care staff) to try to make the environment right for the child, so their autism was less ‘disabling’.

The ratings by the two multidisciplinary groups of health and education professionals, and by the group of ASD researchers, showed low agreement with the averaged ratings of the parent groups (r_s_ = −0.268, 0.131 and −0.063 respectively). The health and education professionals commented that they measure what they can (in the setting, given the available tools) and what they traditionally have done. They emphasized as ‘important’ those things they see as most urgent to try to change, such as challenging behaviour and poor communication skills. In contrast, whilst acknowledging the importance of the construct ‘positive views of self (self-esteem)’, they gave it a lower rating because of the developmental stage of children up to 6 years of age. The researchers also rated self-esteem as ‘low’, but did so due to lack of a suitable measurement tool. The researchers rated highest ‘not cooperating, throwing, spitting, won’t sit (maladaptive behaviour)’ on the basis of its impact on others and on the child’s experience. Both groups of health and education professionals identified a range of additional outcome constructs they would consider important to measure, including communicative competence, problems with food, functional adaptive behaviour, etc. They also mentioned the importance of identifying the skill set of support staff, and parent confidence in managing their child’s needs and behaviours.

When all groups came together, the discussion highlighted differences in their perspectives. The parents and the young adult on the autism spectrum argued that it is important to focus on what children can do, to see autism as a ‘difference’ rather than always using a ‘deficit’ model, and to focus more on how to enable children through improving their environments. Parents were encouraged that the clinicians had mentioned including assessment of the skills of care and education staff. The clinicians reflected that their approach to assessment and intervention is based on the ‘medical’ model: early identification of specific impairments, choice of appropriate treatment, prevention of secondary impairment, and so on. Their measurement of outcomes and the tools available to them reflect this framework, with an emphasis on problems and deficits. For the researchers, their model of intervention and outcome assessment was also primarily embedded in a ‘deficit’ model of autism, with an emphasis on treating and measuring changes in the core features of autism. Research outcomes such as helping parents manage better and understand more would, in their view, be classed as ‘soft outcomes’, and not given the same importance as changing children’s characteristics.

## Discussion

We took a multi-faceted approach to identifying what outcomes parents/carers value when monitoring the progress of young children with ASD. The initial stages included a scoping review of published qualitative literature, and a series of consultation meetings with parent advisory groups. Parents’ prioritisation of outcomes was presented and discussed with health and education professionals and autism researchers.

Parents in the advisory group discussions valued a wide range of outcomes across all domains of their child’s functioning—abilities, difficulties, everyday activity and participation. In summary, parents appeared to focus on what is important in terms of living with ASD on a daily basis, namely on reducing stress and building up necessary skills for future functioning, and on the need for environments to be more ‘autism-friendly’, thereby promoting participation. Their perspective could be identified as reflecting the ‘social model of disability’ (Oliver 2004), as well as acknowledging their child’s difficulties. Inspection of the outcomes measured in longitudinal and early intervention evaluation studies (McConachie et al. 2015) suggests a strong adherence to the ‘medical model’ of clinical practice, with a focus on measuring what children cannot do, assessing symptom characteristics with diagnostic tools (Castro et al. 2013), with treatments targeting reduction in impairments, rather than measuring outcomes appropriate to interventions that are designed to support parents in managing their child’s current behavioural profile and build on their children’s functioning and wellbeing (Smith et al. 2015). Measures focusing on ASD symptoms relate to outcomes which may or may not be amenable to change, and may or may not be related to the focus of interventions (Wolery and Garfinkle 2002; Lord et al. 2005).

In considering what outcomes should be measured, all domains of the ICF framework are potentially relevant. It requires collaboration amongst professionals, and inclusion of parents/carers, to measure each child’s progress. The same collaboration should apply to determining a set of key outcomes to use in research and monitoring of progress that all stakeholders will value, whilst at the same time recognising the goals and constraints of measurement. In future work, consultation between families and professionals might usefully analyse further how the broader goals of parents—for best functioning and participation, and reduction of distress—might be linked with the more specific impairment and disability-focused goals of professionals. The development of a consensus about ‘what are the relevant child outcomes?’ should include consideration of a broad repertoire of behaviours such as social interaction skills (e.g. with brothers, sisters and other children), everyday adaptive skills, recognition of co-occurring problems (e.g. sleep, eating), wellbeing of the child, and family quality of life. Bringing together these different perspectives on valued outcome constructs would be likely to benefit children with ASD and their families, and is consistent with the recommendations of the UK Kennedy Report (Kennedy 2010). It is also consistent with discussions about how to enable evaluation research on early interventions to make the transition into practice, by ensuring that the outcome evidence is valued by families (Dingfelder and Mandell 2011).

### What Outcomes Should be Measured?

In longitudinal observational studies, and in intervention trials, the decision about what outcomes to measure is influenced by a number of considerations. First, there is the question of what should be the primary focus of a longitudinal study, or the primary goal of intervention. This is partly determined by the aims and content of a particular intervention, but also by a decision on whether to focus on reducing particular ASD impairments or overall symptom severity, or improving child functional outcomes, or targeting problem behaviours which are affecting the quality of life of the child and his/her family. Each of these implies different conceptual and practical considerations, a principled choice of primary outcome, and associated measurement tools. Second, there is the question of the merits of measuring ‘specific’ versus ‘general’ outcomes. The value of *specific* outcomes is that they are focused, close to the target of a particular intervention approach and so useful in investigating the efficacy of the intervention (Yoder et al. 2013). However an emphasis on specific targeted outcomes can be misleading in relation to the actual effectiveness of interventions. If observed specific improvements do not generalise, and lead to better functional outcomes, then it is questionable whether the effort and costs attached to such an intervention are justifiable to families and/or commissioners of services. This issue is of particular relevance in the field of evaluation of autism interventions as individuals with ASD have been shown to have considerable difficulty in generalising skills learned in one setting into their everyday functioning (e.g. Cowan and Allen 2007). It is therefore important that clinicians, researchers and parents/carers develop a shared understanding of the likely mechanism of change for young children with ASD, and a more comprehensive and multifaceted theoretical model of skills development, so that the connections between various levels of outcomes can be better understood (Green and Dunn 2008). In addition, more work is required to provide evidence for a reasonable timeframe within which generalisation of skills might be expected to be observed in individuals with ASD; acquisition of new specific skills such as joint attention might be observed within 3–4 months, but generalisation into other settings and interaction partners (i.e., an established functional skill) is likely to take longer. A third, related consideration for measurement has to do with external validity (Jonnson et al. 2015). The dilemma here is that subjective (particularly family-reported) measures are those with the greatest external validity, since it is the experience of children and families that those providing intervention most want to improve; however, such ratings are prone to expectation and placebo effects that need to be controlled for in any rigorous evaluation of an intervention (Valderas and Alonso 2008). Furthermore, the commissioned review revealed a paucity of outcome measurement tools with robust measurement properties in many areas of everyday functioning and participation (McConachie et al. 2015).

We found considerable overlap between the valued outcomes specific to children with ASD and the proposed core suite of outcomes for children with neurodisability generally, namely: communication, emotional wellbeing, pain, sleep, mobility, self-care, independence, mental health, community and social life, behaviour, toileting and safety (Morris et al. 2015). That work also identified a lack of generic multidimensional child and/or parent reported outcome measures available to address many of these outcomes. Future initiatives should focus on producing valid and reliable tools that measure outcomes which include consideration of what matters to people on the autism spectrum and their families.

### Limitations and Strengths

This process of multi-method consultation and review of qualitative literature had a number of limitations. The focus was necessarily narrow with respect of the age range of children, as the commissioned brief was to focus on young children with ASD (up to the age of 6 years). Future consultation would be needed regarding outcome measurement for older children and adults on the autism spectrum, from across the age and ability range.

The strengths of this study include the mixed methods and iterative approach to consultation. We were able to include the views of parents of young children with ASD both with and without intellectual disability and/or limited communication skills and from different parts of the country.

### What Should be the Next Steps?

The consultation has highlighted the importance of engaging parents and adults on the autism spectrum in research processes from the start of when a study is conceived, working with researchers to agree the research questions, understanding the process of research design and together identifying the most appropriate outcomes to measure, and understanding the strengths and limitations of the chosen measurement tools. Consistency and interpretation of evidence about progress and outcomes can be improved if researchers routinely collect and report on an agreed set of core outcomes (Williamson et al. 2012). Initiatives such as COMET (Core Outcome Measures in Effectiveness Trials) are helping to identify best practice in reaching agreement about what should be included in a core set of outcomes in various fields of healthcare (http://www.comet-initiative.org/), alongside the development of a reporting guideline (Kirkham et al. 2015). A process of developing core sets of outcomes for ASD across the life span is underway, albeit this is based solely on ICF components of health (Bolte et al. 2014). The current paper suggests ways to engage parents in working towards a future core set of outcomes to be measured in intervention evaluation for young children with ASD.

## Electronic supplementary material

Below is the link to the electronic supplementary material.

Supplementary material 1 (DOCX 17 KB)
