# Supplementary material for: Parents Suggest Which Indicators of Progress and Outcomes Should be Measured in Young Children with Autism Spectrum Disorder
Source: J Autism Dev Disord. 2017 Aug 31;48(4):1041–51. doi: 10.1007/s10803-017-3282-2 (PMC5861173; doi:10.1007/s10803-017-3282-2)
Supplement: Supplementary file 1 — Supplementary material 1 (DOCX 17 KB) [file 10803_2017_3282_MOESM1_ESM.docx]

*Appendix 1*

Constructs in lay language as presented to parent groups (range -5 to +5), organized in ICF domains of basic abilities/impairments, activity level, participation and family outcomes. (In parentheses, the ICF terms or research terms.)

| **Domain** | **Basic abilities / impairments**  (Body functions and structure) | |
| --- | --- | --- |
| **Subdomain** | **Construct** | **Mean Rating of Importance** |
| Awareness of other people | Looking at object and other person  (Joint attention skills) | -1.25 |
|  | Copying other people  (Imitation) | -1.00 |
|  | Picking up social cues, making eye contact (Social attention) | -0.05 |
| Repetitive behaviour | Repetitive and unusual movements | -2.75 |
|  | Repetitive use of objects | -4.00 |
|  | Unusual attention to detail and specific interests | -1.75 |
|  | Rigid routines; repetitive language (Insistence on sameness) | -0.50 |
| Sensitive to stimulation  *(sensory processing)* | Discomfort with being touched, too much noise, bright lights, certain tastes, etc. (Hypersensitivity) | 2.75 |
|  | Not feel pain, seek out noisy environment, drawn to strong smells, etc. (Hyposensitivity) | -0.75 |
| Language | Amount of speech; complexity of speech; speaking clearly  (Expressive language) | 1.75 |
|  | Listening and understanding what others say  (Receptive language) | 1.50 |
|  | Other conventional ways of communication  (Gestures) | 0 |
| Intelligence  *(cognitive ability)* | Capacity for reasoning and understanding compared to the general population  (I.Q.) | 1.25 |
|  | Understand visual information and solve problems using visual reasoning  (Nonverbal ability) | 2.25 |
|  | Able to listen to and remember spoken information. Work out categories (eg a cat is an animal)  (Verbal ability/reasoning) | -0.50 |
| Attention | Easily distracted, short attention  (Distractibility) | 0 |
|  | Impulsive behaviour | -1.50 |
|  | Overactive, fidgety  (Hyperactivity) | -1.75 |
| Emotional state (Self-regulation) | Happiness | 3.75 |
|  | Irritability | -0.25 |
|  | Distress | 2.50 |
|  | Anxiety, unusual fears | 3.50 |
| Physical coordination | Difficulty walking, running, balancing  (Gross motor skills/poor coordination) | -2.00 |
|  | Hand and finger skills, hand-eye coordination, drawing, writing  (Fine motor skills) | 0 |
| **Domain** | **Learning, Behaviour and Skills** (Activity level) | |
| **Subdomain** | **Construct** | **Mean Rating of Importance** |
| Social communication | Approaching others, beginning interactions  (Quality/frequency of initiations) | -1.75 |
|  | Taking turns in communicating  (Pragmatics) | 0.25 |
| Social functioning | Attachment, e.g. using parent as ‘safe place’ | 0.75 |
|  | Interaction skills with other children | 1.00 |
|  | Awareness of other's emotions; reading facial expressions | -1.00 |
| Play | Level of play (from simply handling a toy, through to pretending) | -3.25 |
|  | Able to organise own time/activities | -3.50 |
| Behaviour | Not co-operating, throwing, spitting, won’t sit, etc.  (Maladaptive behaviour) | -0.75 |
|  | Fighting, hitting others  (Aggression) | 2.00 |
|  | Tantrums, melt-downs | 0.75 |
|  | Self-injury | 0.75 |
| Habit problems | Long time to fall asleep; wake up in night  (Sleep problems, latency and waking) | 2.00 |
|  | Picky eating; eat too much, too little; physical problems in eating; behaviour problems interfering with eating  (Eating problems) | -0.50 |
|  | Not toilet trained, smear poo, etc  (Toilet problems) | -0.75 |
| Learning | Ready to learn from school curriculum  (School readiness) | 1.50 |
|  | Interest in letters, books; early reading  (Early literacy) | -0.75 |
|  | Interest in numbers and quantities  (Early numeracy) | -1.50 |
| Daily living skills | Feeding self using cutlery | -3.00 |
|  | Dressing self | -2.75 |
| **Domain** | **Wider Social Relationships and Quality of Life** (Participation) | |
| **Subdomain** | **Construct** | **Mean Rating of Importance** |
| Social relationships | Relationships with brothers and sisters | 2.25 |
|  | Friendships | -0.25 |
|  | Attending family events | -1.00 |
|  | Attending birthday parties and social events | -1.75 |
| Subjective wellbeing  (Quality of life) | Coping/resilience | 1.00 |
|  | Positive views of self  (Self-esteem) | 2.75 |
| Social inclusion | Participates in mainstream activities  (Social inclusion) | -0.50 |
|  | Experiences rejection by others; is bullied  (Social exclusion) | 2.00 |
|  | Difficulty attending appointments (e.g. vision check, haircut), having feet measured, etc. | 0 |
|  | No awareness of danger; vulnerable. | 1.75 |
| **Domain** | **Family** | |
| **Subdomain** | **Construct** | **Mean Rating of Importance** |
| Interaction style | Parent responds to child communications  (Synchrony) | 1.25 |
|  | Parent joins in with child activity  (Shared attention) | 1.00 |
| Parenting behaviours/ style | Parent firm and fair | -3.00 |
|  | Parent warmth to child | 1.50 |
| Parent stress | Stress (body symptoms, poor sleep, etc) | 2.25 |
|  | Coping style (e.g. problem solving, ‘wait & see’) | 0 |
|  | Parent anxiety or depression | -0.25 |
| Family quality of life | Family sticking together, helping each other  (Family cohesion) | 1.00 |
|  | Breakdown in family relationships, or increased family resilience  (Impact on family) | 0.75 |
